# Pathway insights: exploring profiles and processes of drop-in students in relation to completion rates in second chance education

**DOI:** 10.3389/fpsyg.2025.1620330

**Published:** 2025-11-28

**Authors:** Lana Van Den Berghe, Sarah S. W. De Pauw, Stijn Vandevelde

**Affiliations:** Department of Special Needs Education, Ghent University, Ghent, Belgium

**Keywords:** Second Chance Education, school dropout, basic psychological needs, educational motivation, self-determination theory

## Abstract

**Introduction:**

Obtaining an upper secondary degree is highly esteemed for its economic and societal value. For students dropping out of school who return to education to earning an upper secondary degree (cf. high school—ISCED level 3)—termed “drop-in students” in this study—alternative pathways like “Second Chance Education” (SCE) have emerged. Empirical studies on these programs are limited, hindering theoretical progress and the understanding of drop-in students’ psychological and educational processes. To address this, the study aims to examine the profiles of drop-in students and the processes that may influence course completion rates in SCE. In doing so, it contributes to the growing body of research on SCE by investigating (1) student profiles and (2) the processes potentially affecting completion rates within a large sample of drop-in students.

**Methods:**

On this basis, a novel model was developed, including background variables (demographic, educational, and employment information), individual processes (educational motivation, aspirations, psychological needs, and wellbeing), and environmental processes (supportive relationships and contextual barriers). Conducted in Belgium, this quantitative study surveyed 528 drop-in students (M_*age*_ = 25; 58% male).

**Results:**

The results advance theoretical understanding and offer insights into the profiles of drop-in students and the processes influencing completion rates (i.e., lower completion rates for those students who speak a different home language, experienced grade retention, have lower motivational quality, higher relatedness and competence frustration, and fewer supportive relationships).

**Discussion:**

This study points to implications of reimagining support for students in education, emphasizing the need for a comprehensive approach to address various aspects simultaneously.

## 1 Introduction

In most societies to date, achieving an upper secondary educational degree (cf. high school—ISCED level 3) is economically and societally perceived as a valued goal in life (Nada et al., 2020; Kiprianos and Mpourgos, 2025; Bangquiao and Galigao, 2025). In Europe, the number of students not attaining this goal, and thus dropping out of upper secondary education (cf. high school—International Standard Classification of Education (ISCED) level 3 (UNESCO, 2011), is expected to increase in the coming years, partly due to the aftermath of the COVID-19 pandemic, associated temporary school closures, and resulting learning gaps (Eurostat, 2022). Many studies, however, pointed out that dropout is the result of a cumulative process of negative school experiences, based on a myriad of reasons at individual and environmental levels (Gren-Landell, 2021; Havik and Ingul, 2022; Van Den Berghe et al., 2022c), and that unfavorable consequences affect people’s life course (e.g., higher rates of unemployment, poverty, juvenile crime, single parenthood) (Archambault et al., 2022; Profiroiu et al., 2024), and society (e.g., political and social apathy, and lost tax revenues from unemployment) (Finning et al., 2022; Ban and Costin, 2025).

Globally, “Second Chance Education” (SCE) has emerged as an alternative pathway for students who dropped out before earning an upper secondary degree (cf. high school—ISCED level 3) (European Commission et al., 2015; Karakitsiou et al., 2024). Despite differences in organization, SCE programs share the goal of re-engaging dropouts in education (Kiprianos and Mpourgos, 2020; McGregor et al., 2015). Though still under-researched, SCE shows promising effects, offering students a “window of opportunity” for personal growth, skills development, and future prospects (Van Den Berghe et al., 2024b; Masten, 2014). However, scientific knowledge on SCE remains scarce. One challenge is the lack of a clear term for those returning to education after dropping out. This study adopts “drop-in students” to describe this group (Van Den Berghe et al., 2024a). While some European countries have issued policy reports, empirical research, especially on motivational and educational processes and completion rates, is limited (Kelly et al., 2022). Therefore, we address a significant gap in this largely underexplored research topic by examining (1) the profiles of drop-in students in SCE and (2) the processes linked to their course completion.

Due to the limited research on this topic, we developed a novel model based on three key assumptions. First, completion rates in SCE are like to be linked to educational inequalities rooted in student backgrounds (Kearney et al., 2022; Ingul et al., 2019; Rabelo and Zárate, 2024). Second, similar to dropout, drop-in processes are best understood through a social-ecological lens, capturing the interplay of individual and environmental factors (Van Den Berghe et al., 2022c; Gren-Landell, 2021). Third, mechanisms influencing school completion in first chance and adult education are also relevant to SCE.

Guided by these assumptions and prior research, we identified key constructs at the “background,” “individual,” and “environmental” levels that are likely to influence drop-in completion. At the level of “background characteristics,” we delved into governmental reports and scientific research and selected the next constructs potentially influencing drop-in processes: (1) demographic characteristics, (2) educational background information, (3) employment information, and (4) SCE-related information. First, dropout research highlights “demographic factors,” such as age, gender, socioeconomic status (Muthuswamy, 2023; Moreno et al., 2025; Martín-Arbós et al., 2024), home versus instructional language (Lopez-Agudo et al., 2021), and parental background (OECD, 2017), as major key predictors of dropout. Second, in SCE, “educational background” factors like prior educational track (e.g., general education, vocational education, special needs education, etc.) (Eurostat, 2023; Gubbels et al., 2019), and the highest degree attained upon dropping out (i.e., the dropout-timing in the student’s educational pathway) (Flemish Department of Education and Training, 2009) are important, as later dropouts may have fewer study requirements in SCE. Lastly, grade retention—also known as grade repetition or being held back—also strongly predicts underachievement and dropout (Anderson et al., 2019; Banaag et al., 2024). Third, “employment information,” such as work status (Goodman et al., 2021) and financial pressures (Portela Pruaño et al., 2022), may create tension between pursuing SCE and entering the workforce (Van Den Berghe et al., 2024a; Kelly et al., 2022). Fourth, “SCE-related information” like enrollment type, study track, number of modules, and recognition of prior competencies are also linked to completion (Flemish Department of Education, 2022; Nada et al., 2020).

At the “individual level,” dropout research often focuses on “intrapsychological processes,” with “Self-Determination Theory” (SDT) widely used to study student motivation, personality, and wellbeing (Ryan, 2023). SDT links educational aspirations (i.e., intrinsic and extrinsic goal orientation) to the satisfaction of basic psychological needs (i.e., autonomy, relatedness, competence) (Veiga et al., 2014; Cret, 2025), which, in turn shape motivation quality and wellbeing (Hope et al., 2019; Vansteenkiste et al., 2020). Based on this, we identify four key constructs influencing drop-in processes: (1) educational motivation, (2) educational aspirations, (3) psychological basic needs, and (4) general wellbeing (Bargmann et al., 2022). First, “educational motivation” is a central concept in educational psychology, closely linked to student achievement (Ryan, 2023; Urhahne and Wijnia, 2023). Motivation involves goal pursuit, engagement, and response to instruction (Anttila et al., 2023). In adult education, learners tend to be more extrinsically than intrinsically motivated (Kalenda and Kocvarova, 2021). Motivation is also a key predictor of course completion in SCE (Christodoulou et al., 2018). Second, in first chance education, “educational aspirations” play a crucial role in student engagement and dropout prevention, with intrinsic aspirations linked to better outcomes (Jeno et al., 2018; Koludrović and Ercegovac, 2023; Pellegrini et al., 2025). In SCE, students often pursue education for extrinsic reasons like employment or self-development (Kiprianos and Mpourgos, 2020; Gardner et al., 2022), although some show strong aspirational capital, boosting achievement (Daniels, 2020). Third, “basic psychological needs” —autonomy, relatedness, and competence—strongly influence success in regular education and protect against disengagement (Hope et al., 2019). In SCE, only one study explored these needs, calling for a supportive climate that fosters autonomy and belonging (Gueta and Berkovich, 2021). Fourth, “student wellbeing” is widely recognized as a buffer against failure and dropout in first chance education (Morelli et al., 2023; Van Den Berghe et al., 2024b). Wellbeing enhances both attendance and life satisfaction (Koludrović and Ercegovac, 2023; Morelli et al., 2023). Although limited, SCE research confirms the link between motivation and wellbeing (Portela Pruaño et al., 2022).

Next to background characteristics and individual variables, we also put “environmental,” research-based constructs that may influence dropout and school engagement in SCE: (1) supportive relationships and (2) contextual barriers. First, “supportive relationships,” including emotional connection and relatedness with family, teachers, and others, are key to educational perseverance (Van Den Berghe et al., 2022a; Piscitello et al., 2022). In SCE, family encouragement (Sagna and Vaccaro, 2022; Bilar and Montañez, 2024), and warm teacher student relationships (Hickey et al., 2020) are especially important. Second, a mix of educational, financial, and family factors can either *push or pull* students back into or out of education (Portela Pruaño et al., 2022), potentially acting as “contextual barriers” toward education (Van Den Berghe et al., 2024b). These decisions involve ongoing balancing between personal aspirations and life demands like parenting, housing, or school distance (Keita and Lee, 2022).

Taken together, this study aims to contribute to the burgeoning research on SCE by exploring (1) the profiles and (2) the processes potentially related to completion rates of courses in a large group of drop-in students. In line with the assumption that similar mechanisms influencing school dropout will also impact school drop-in in SCE, our study model will scrutinize the impact of variables at the *background* (i.e., demographic information, educational background, employment information, and SCE-related information), *individual* (i.e., educational motivation, educational aspirations, basic psychological needs, and general wellbeing), and *environmental* (i.e., supportive relationships and contextual barriers) levels.

## 2 Materials and methods

### 2.1 Research setting and participants

This study was conducted in Flanders, the Northern part of Belgium, a region responsible for its educational policy with 6.5 million inhabitants (Statistics, 2023). In Flanders, statistics on the school administrative data summarizes that in 2022, 14.1% of students dropped out of school, with a higher risk for students with grade retention, students in vocational training programs, and students who speak another language at home than the language of instruction (Statista Research Department, 2024). The most recent data on SCE dates back to 2020 state that in Flanders, about 20.2% of the dropout students re-enroll in SCE (Flemish Department of Education, 2022). This study took place between September 2022 and December 2023, in the aftermath of the COVID-19 pandemic. Data was gathered from 528 drop-in students in 13 schools for SCE, across 22 campuses in Flanders (see Table 1).

**TABLE 1:** Overview of the participating SCE schools.

School	N campuses	N participants	City	Data collection	Study track options SCE
1	1	77	Large city	In classes	General + vocational
2	2	25	Small + medium city	In classes	Vocational
3	3	40	Small + medium city	In classes	Vocational
4	1	45	Large city	In classes	Vocational
5	1	25	Small city	Digital	Vocational
6	1	9	Large city	In classes	Vocational
7	1	43	Large city	In classes + digital	General + vocational
8	1	32	Large city	In classes + digital	General + vocational
9	1	24	Small city	In classes + digital	Vocational
10	1	32	Small city	In classes	Vocational
11	3	113	Small cities	In classes	General + vocational
12	3	50	Small + large city	In classes	General + vocational
13	2	13	Small + medium city	In classes	Vocational

### 2.2 Research design

Before this study, a pilot study (*N* = 40) was conducted in a school for SCE in the Ghent area. In this pilot, we thoroughly tested the questionnaire in terms of comprehensibility and readability for the drop-in students, assessing the task load and reliability of questionnaires and scales. The Cronbach’s alpha’s were considered to be sufficient for all measured constructs (i.e., > 0.70 (Taber, 2018)). When the understanding of words was challenging for participants, the validated questionnaires were still used, but synonyms of these words were added to the items (cf. the Dictionary of Dutch Language).

**FIGURE 1:**
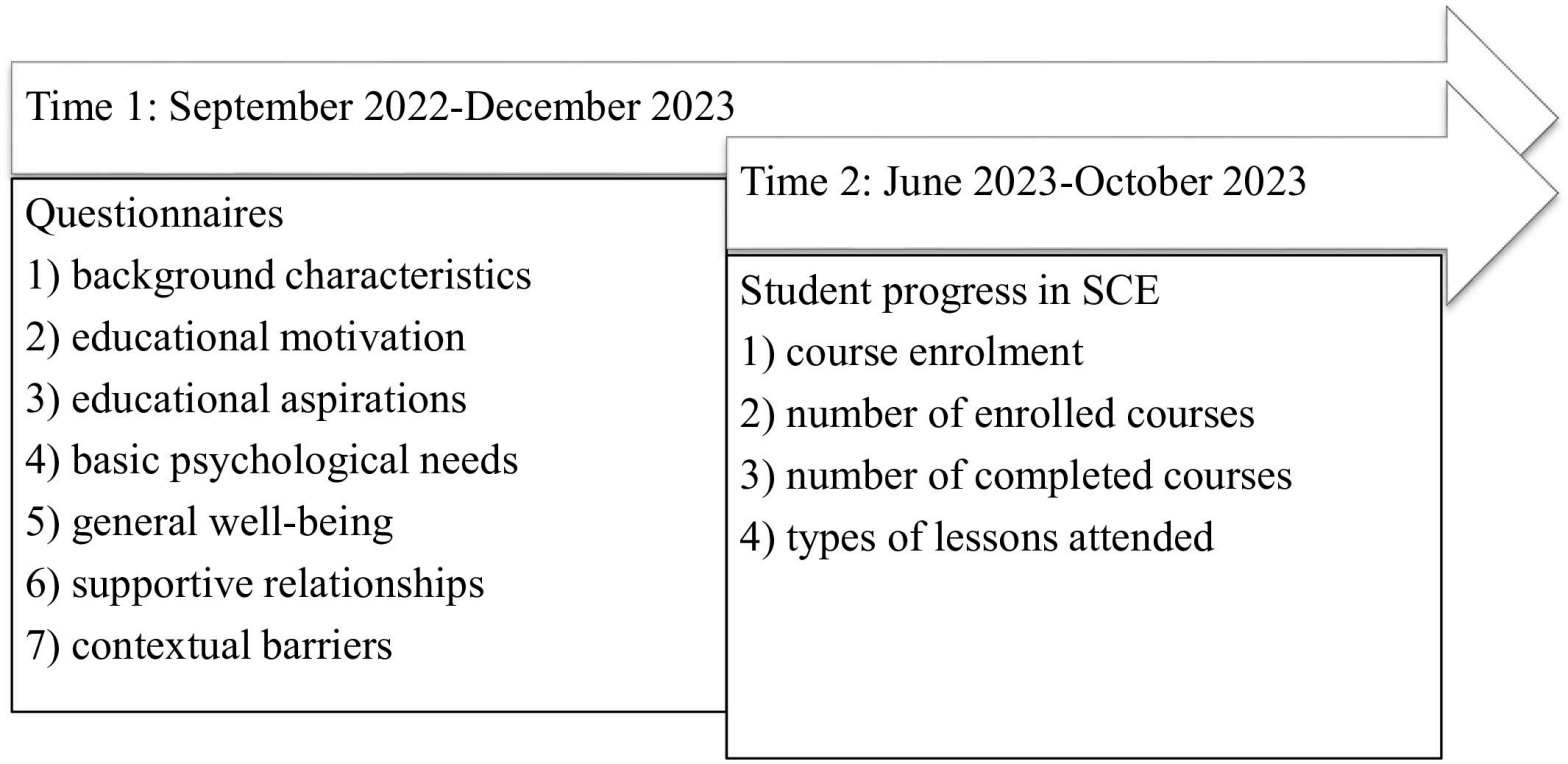
An overview of the phases of this study.

Based on the pilot study, the full study was conducted, divided into two time periods. Figure 1 provides an overview of the constructs measured in each period. Time 1 included data collection using digital questionnaires, in Time 2, the study progress in SCE of the participants was linked to the data of Time 1.

Time 1 took place between September 2022 and December 2023. During this time, the students filled in a digital questionnaire covering the different constructs of interest: (1) background characteristics (i.e., demographic information, educational background, employment information, and SCE-related information), individual (i.e., (2) educational motivation, (3) educational aspirations, (4) basic psychological needs, and (5) general wellbeing), and environmental levels (i.e., (6) supportive relationships and (7) contextual barriers). Teachers and support workers from the participating schools conducted the data collection by administering the questionnaires during lessons, through the digital school platform, or by using a combination of both. The median duration of the questionnaires was 24 min (*IQR* = 18 min, 36 min).

Time 2 took place between June 2023 and October 2023. During this time, information was gathered about students’ educational progress in SCE in the year of participation in this study. We coded: (1) enrolled study option, (2) number of enrolled courses, (3) number of completed courses, and (4) type of lessons attended. This information was provided by the teachers and support workers of the participating schools and was subsequently linked to the data of Time 1. We were able to attain these data for 95.27% (*N* = 504) of the original sample.

### 2.3 Overview of research variables and instruments

#### 2.3.1 Dependent variable

This study aimed to investigate the profiles and processes potentially related to completion rates of courses of drop-in students in SCE. Therefore, we chose “completion rates” in SCE as the dependent variable of this study. Choosing this variable presented us with some challenges because the SCE programs are primarily designed to be short-term, typically spanning approximately 2 years. However, the exact length may vary depending on the previously gained knowledge and competencies in regular (first chance) education, and the specific courses a student needs to complete to be eligible to gain his/her degree. In Flanders, SCE is open to adults aged 18 and above, and employs a modular approach (cf. courses), allowing students to choose flexible learning trajectories, that include the option to combine both in-person and distance learning courses. Overall, each student has a unique and flexible educational program to complete, making it challenging to tell how “successful” students are in completing courses during their SCE program, and thus to provide objectively measured outcomes of completion of SCE trajectories in this study.

**FIGURE 2:**
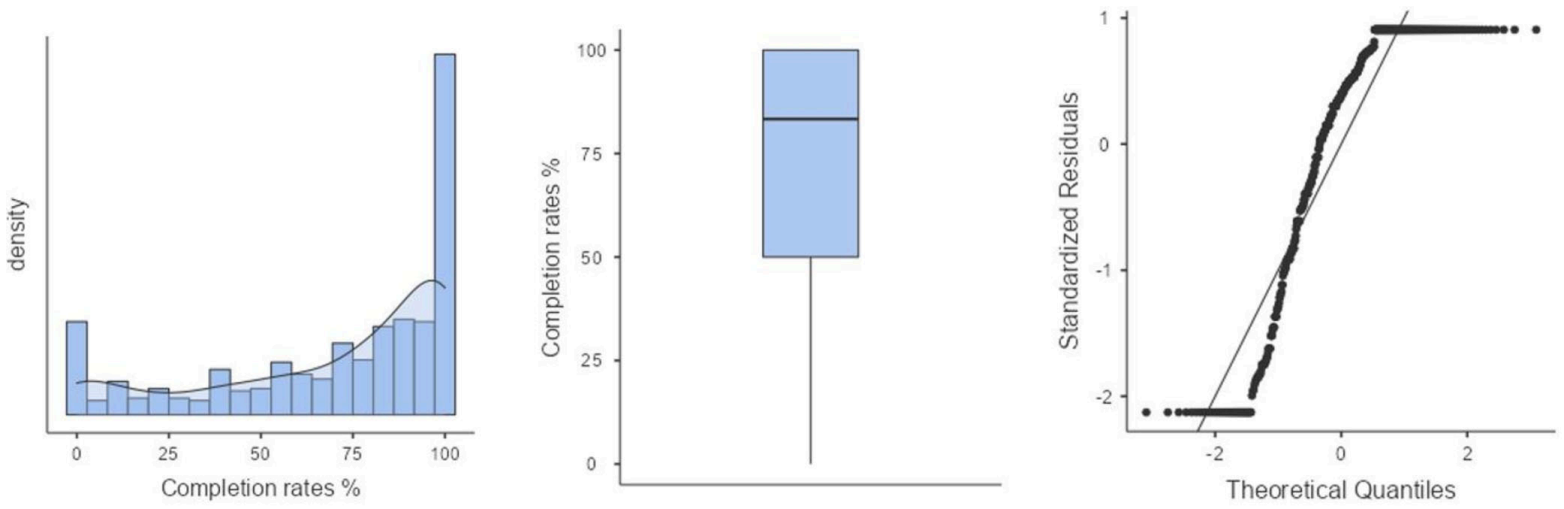
Visualization of the distribution of the dependent variable completion rates (%).

To address this, the teachers and support workers of the various schools counted the number of enrolled courses of each participant (*Mdn* = 13; *IQR* = 8–18) as well as the number of completed courses (*Mdn* = 9; *IQR* = 5–14), both in the academic year of participation in this study. The “completion rates (%)” were calculated based on the number of modules completed relative to the total number of modules enrolled in the academic year of participation (*Mdn* = 83.33; *IQR* = 50.00–83.33%). The distribution of this variable revealed a group of participants who did not complete a single module, as well as a large group who passed all modules (see Figure 2). Based on this observation, we proceeded to examine the percentiles in the distribution of this variable, leading us to decide to use the percentiles of “completion rates (%)” as cut-off scores to divide the sample into three groups: Group 1—“low completion rates group” (*N* = 162; completion rates ≤ 50.00%), Group 2—“medium completion rates group” (*N* = 89; completion rates > 50.00% and ≤ 83.33%), and Group 3—“high completion rates group” (*N* = 253; completion rates > 83.33%).

#### 2.3.2 Independent variables

Based on our literature review, we selected the following variables on the background, individual, and environmental level.

“Background characteristics” were addressed using self-designed questions, based on recent literature (Lawrence and Adebowale, 2023), the most recent statistics on school dropout profiles (Eurostat, 2023; Statbel, 2023), and the existing reports on profiles of students in SCE (Flemish Department of Education and Training, 2009). Four categories of background variables were selected: (1) demographic information, (2) educational background information, (3) employment information, and (4) SCE-related information (see Table 2 for an overview).

**TABLE 2:** An overview of the background characteristics of the participating drop-in students in SCE.

Background characteristics	Categories	Frequencies
**Demographic information**		
Age groups	≤ 25 years of age 26–35 years of age 36–45 years of age >45 years of age	374 (71.0%) 072 (13.7%) 046 (8.7%) 035 (6.6%) *N* = 527
Gender	Men Women	300 (57.7%) 220 (42.3%) *N* = 520
Language spoken at home	Dutch Other languages	319 (60.4%) 209 (39.6%) *N* = 528
Country of birth	Belgium Other	341 (64.6%) 187 (35.4%) *N* = 528
Highest gained degree mother (cf. according to ISCED)	Primary education (i.e., ≥ 2th grade Secondary Education) Secondary education (i.e., ≥ 6th grade Secondary Education) Tertiary education (i.e., higher education)	064 (24.7%) 106 (40.9%) 089 (34.4%) *N* = 259
Highest gained degree father (cf. according to ISCED)	Primary education (i.e., ≥ 2th grade Secondary Education) Secondary education (cf. ≥ 6th grade Secondary Education) Tertiary education (cf. higher education)	041 (20.7%) 083 (41.9%) 074 (37.4%) *N* = 198
**Educational background**		
Highest gained degree before school dropout (cf. according to ISCED)	Primary education (i.e., ≥ 2th grade Secondary Education) Secondary education (i.e., ≥ 6th grade Secondary Education) Tertiary education (i.e., higher education)	350 (66.3%) 161 (30.5%) 017 (3.2%) *N* = 528
Study track during school dropout	Outside Belgium General education Vocational education Special needs education	084 (15.9%) 283 (53.6%) 48 (9.1%) 0113 (21.4%) *N* = 528
Grade retention before school dropout (i.e., “blijven zitten”)	Yes 1 time 2 times 3 times 4 times No	267 (50.7%) 159 (29.9%) 78 (14.8%) 20 (3.8%) 4 (0.8%) 260 (49.3%) *N* = 527
Age of school dropout	≤ 16 years of age 17-18 years of age 19-20 years of age ≥ 21 years of age	81(15.4%) 290 (55.0%) 115 (21.8%) 041 (7.8%) *N* = 527
**Employment information**		
Employment experience	Yes No	427 (86.6%) 66 (13.4%) *N* = 493
Current type of employment upon participation	Work Student employment No work	168 (34.1%) 187 (37.9%) 138 (28.0%) *N* = 493
Current financial income upon participation	Work Unemployment allowance Disability allowance No income	156 (31.6%) 105 (21.3%) 036 (7.3%) 196 (39.8%) *N* = 493
**SCE-related information**		
Chosen course in SCE	Vocational oriented education General education	475 (94.1%) 030 (5.9%) *N* = 505
Type of lessons in SCE	Classroom learning Distance learning modules Combination	358 (71.0%) 028 (5.6%) 118 (23.4%) *N* = 504
Exemption for a course module in SCE	Yes No	397 (78.8%) 107 (21.2%) *N* = 504

The seven domains that emerged from our literature review (i.e., at the “individual level”: (1) educational motivation, (2) educational aspirations, (3) psychological basic needs, and (4) general wellbeing: at the “environmental level”, (5) supportive relationships, and (6) contextual barriers) were measured based upon a carefully constructed set of questionnaire scales, chosen by their validity and the availability of a Dutch version of the questionnaires. An overview of these scales can be found in Table 3.

**TABLE 3:** Overview of the constructs chosen with the related independent variables, instruments, items, and Cronbach’s Alpha’s.

Place in our model	Constructs chosen	Independent variables	Instruments	Items	Cronbach’s alpha
Background characteristics	Background characteristics	Demographic information Educational background Employment information SCE-related information	Self-made questions	15	
Individual processes	Educational motivation	Autonomous motivation Controlled motivation Amotivation	Organismic Motivation Continuum, Self-regulation questionnaire (Vansteenkiste et al., 2009)	20 items 5-point Likert-scale ^a^	α = 0.89 α = 0.84 α = 0.84
	Educational aspirations	Intrinsic aspirations Extrinsic aspirations	Aspiration Index—Dutch version (Duriez et al., 2007)	15 items 5-point Likert-scale ^a^	α = 0.79 α = 0.88
	Basic psychological needs	Autonomy satisfaction Autonomy frustration Relatedness satisfaction Relatedness frustration Competence satisfaction Competence frustration	Basic Psychological Need Satisfaction and Frustration Scale—Dutch version (Van der Kaap-Deeder et al., 2020)	24 items 5-point Likert-scale ^a^	α = 0.70 α = 0.83 α = 0.79 α = 0.81 α = 0.72 α = 0.82
	General wellbeing	General wellbeing	Personal Wellbeing Index—Dutch version (van Beuningen and de Jonge, 2011)	8 items 5-point Likert-scale ^a^	α = 0.89
Environmental processes	Supportive relationships	Supportive relationships	Self-made question: “How much support for your study in SCE are you provided by: parents, partner, children, friends, and significant others”	5 items 5-point Likert-scale ^a^	α = 0.84
	Contextual barriers	Contextual barriers	“Personal and Contextual Factors” subscale of the VASEV-LL—Dutch version (Depreeuw et al., 2010)	7 items 5-point Likert-scale ^a^	α = 0.79

*^a^*The 5-point Likert-scale range: 1 = completely disagree, 2 = disagree, 3 = neither disagree nor agree, 4 = agree, 5 = completely agree.

### 2.4 Data analysis and report

The data analysis was conducted using R-studio with the “CaviR package” (Waterschoot, 2024), and SPSS. The preparation for data analysis consisted of three phases. First, an dependent variable “completion rates groups” was computed based on the percentiles of “completion rates (%).” Second, the selected background characteristics were coded into categorical independent variables (see Table 2). Third, the reliabilities of the different items and questionnaires used in this study were tested to determine how much of the variability in test scores is due to variability in the scores. The Cronbach’s alpha’s were considered to be sufficient when > 0.70 (Taber, 2018) (see Table 3).

The analyses to answer the research questions consisted of four tests. To answer the first aim of this study (i.e., identifying profiles of drop-in students), (1) descriptive statistics and crosstabs were used. To answer the second aim of this study, (2) a Chi-Squared test of Independence was used to explore the relationship between the groups of categorical independent variables (i.e., the background characteristics) and the completion rates groups. (3) Pearson correlations were used to explore the correlation between the continuous independent variables (i.e., constructs: educational motivation, educational aspirations, basic psychological needs, general wellbeing, supportive relationships, and contextual barriers) and the completion rates groups. Finally, (4) a one-way MANOVA and subsequent ANOVAs were used to determine whether there was a difference between the completion rates groups for the continuous independent variables.

### 2.5 Ethical considerations

This research was approved by the Ethical Committee of the Faculty of Psychology and Educational Sciences of Ghent University. Informed Consent was used in the questionnaire to inform students about their rights and to ask permission for confidential data processing and anonymous representation. The data that support the findings of this study are available from the corresponding author, L.VDB. upon reasonable request.

## 3 Results

This study aims to contribute to the burgeoning research on second-chance education by exploring (1) the profiles and (2) the processes potentially related to completion rates of courses in a large group of drop-in students.

### 3.1 RQ1: what are the profiles of drop-in students?

To answer this research question, we focussed on the background characteristics of the participants, including demographic information, educational backgrounds, employment information, and SCE-related information, where descriptive statistics and crosstabs were used to explore the data.

First, regarding “personal information,” out of 528 participants, the largest group consisted of students younger than 25 years of age (*N* = 374, 71.0%). In total, 520 students reported their gender, indicating there are 14.6% more men (*N* = 300, 57.7%) than women (*N* = 220, 42.3%) in our sample. The majority of the drop-in students spoke mainly Dutch at home (*N* = 319, 60.4%) which is also the instructional language of education in Flanders. Further, the majority of participants were born in Belgium (*N* = 341, 64.6%), and other participants were born outside Belgium (*N* = 187, 35.4%). In total, 45 different nationalities were reported, including a large group of students with one nationality (*N* = 414, 78.4%), a group with two nationalities (*N* = 108, 20.5%), and a group with three nationalities (*N* = 6, 1.1%). Information about the educational level of parents was only available in 49% (mothers) and 38% (fathers) of the sample, as many participants omitted this question or indicated they did not know. There was a similar, yet widely dispersed range of educational levels in these reports: primary education (cf. according to ISCED) was the highest attained degree of 24.7% of mothers and 20.7% of fathers, secondary education was the highest degree of 40.9% of mothers and 41.9%, whereas a degree of tertiary education was attained by 34.4% of mothers and 37.4% of fathers.

Second, concerning “educational information” about previous school dropout of these drop-in students, the largest group attained their highest degree in “Primary Education” (*N* = 350, 66.3%) (cf. according to ISCED, which is in Belgium ≥ 2nd-grade Secondary Education), while a smaller group achieved their highest degree in “Secondary education” (*N* = 161, 30.5%), only 3.2% of the participants attained their highest degree in “Tertiary education” (*N* = 17). These individuals are mainly students who earned a degree outside Belgium, which is not recognized within the Belgian education system. Concerning the educational tracks students were enrolled in when dropping out of school, the largest group (*N* = 283, 53.6%) indicated that they pursued a study track in “General Education” (i.e., academic-oriented education) at the moment they dropped out from school. Notably, the second largest group of drop-in students were previously enrolled in Special Needs Education (*N* = 113, 21.4%). The third largest group received prior education outside Belgium (*N* = 84, 15.9%). The smallest group (9.1%) consisted of students who were enrolled in vocational tracks in education. Notably, 50.7% of drop-in students reported that they experienced grade retention before school dropout (*N* = 267), including students who dropped out one time (*N* = 158), two times (*N* = 78), three times (*N* = 20) and four times (*N* = 4), with the largest group dropping out at the age of 17-18 years old (*N* = 290, 55%).

Third, “employment information” provided insights into the former work experiences of drop-in students before enrolling in SCE, with the majority reporting work experience (*N* = 427, 86.6%), predominantly in student employment (*N* = 187, 37.9%) and regular work settings (*N* = 168, 34.1%). The majority of drop-in students had no steady financial income at the moment of participating (*N* = 196, 39.8%). However, a considerable number of students combined their SCE study with work and earned income through employment (*N* = 153, 31.6%).

Fourth, “SCE-related information” provides insights into the educational pathways of the participants in SCE itself. One important observation is that the large majority of drop-in students in this study opt for the vocational track in SCE (*N* = 475, 94.1%). This may have to do with the fact that not all SCE schools in Flanders organize a general education track (only 5 of 13 schools in our sample) while all schools offer vocational tracks. Most students (71%) also opt for classroom-attended learning. Only a very small subset opts exclusively for the remote learning modules (5.6%), even though a substantial group chooses a hybrid track, combining in-class and distance learning classes (23.4%). Notably, the majority of drop-in students follow an individual curriculum and set of course modules. Depending on the level of education completed before enrolling in SCE, students have the opportunity to take a test for certain SCE modules. Upon successful completion of the test, students earn credits for the module, granting them waivers for the subsequent course requirements. The vast majority of participants have these “exemptions” or “waivers” for at least one of the courses (*N* = 397, 78.8%).

### 3.2 RQ2: which processes are potentially related to completion rates of drop-in students in SCE?

To answer this research question, different constructs of interest were measured: (1) background characteristics (i.e., demographic information, educational background, employment information, and SCE-related information), individual (i.e., (2) educational motivation, (3) educational aspirations, (4) basic psychological needs, and (5) general wellbeing), and environmental (i.e., (6) supportive relationships and (7) contextual barriers) levels. Three tests were used to explore these processes: (A) a Chi-Squared test of Independence, (B) Pearson correlations, and (C) a one-way MANOVA and subsequent ANOVA’s.

#### 3.2.1 Group differences in categorical background variables

A series of Chi-Squared Tests of Independence was conducted to determine group differences in the categorical independent variables (i.e., all coded background characteristics) and the completion rates groups. Two tests were significant at the *p* < 0.05 level. First, the language spoken at home significantly varied across the three groups, χ^2^(2, *N* = 504) = 12.378, *p* = 0.002. Group 3 (high completion rates) had the highest proportion of Dutch-speaking students (*N* = 163, 64.4%), compared to the other two groups. Second, the proportion of students who reported grade retention before school drop-out also significantly differed across groups, χ^2^(2, *N* = 503) = 6.099, *p* = 0.047. Group 1 (*N* = 90, 55.9%) and 2 (*N* = 48, 53.9%) had significantly more students with grade retention than Group 3 (*N* = 112, 44.3%). Tables 4, 5 present the outcome of the significant tests.

**TABLE 4:** Chi-squared test of independence with completion rate by language spoken at home.

Completion rates	Dutch (304)	Other (200)	Total	χ^2^	df	*p*	Cramer’s V
Low	102	60	162	12.378	2	0.002	0.157
Medium	39	50	89				
High	163	90	253				

Test significant at the *p* < 0.05 level.

**TABLE 5:** Chi-squared test of Independence with completion rate by grade retention.

Completion rates	No grade retention	Grade retention	Total	χ^2^	df	*p*	Cramer’s V
Low	71	90	161	6.099	2	0.047	0.110
Medium	41	48	89				
High	141	112	253				

Test significant at the *p* < 0.05 level.

#### 3.2.2 Correlations between completion rates and the independent variables

Pearson correlation tests were used to measure the continuous completion rates variable on the one hand, and our selected set of individual and environmental variables on the other. In this study, Cohen’s guidelines for interpreting correlation coefficients were adopted, as they are widely used in psychology and facilitate cross-study comparisons (Gnambs, 2023; Lovakov and Agadullina, 2021). Given that human behavioral data typically exhibit substantial variability, correlations of approximately *r* = < 0.10 are considered low, *r* = 0.10–0.30 are considered medium, and *r* > 0.30 are considered high in this study (Cohen, 1988). Table 6 presents the descriptives and Pearson correlations.

**TABLE 6:** Descriptives and correlations between the continuous completion rate variable (%) and the continuous individual and environmental variables.

Variables	M	SD	1	2	3	4	5	6	7	8	9	10	11	12	13	14
1. Completion rates (%) ^a^	70.11	32.96	0.12**	−0.13**	0.54***	−0.16***	0.53***	0.20***	−0.21***	-.16***	−0.32***	−0.19***	−0.31***	−0.38***	0.35***	−0.22***
2. Autonomous motivation	3.6	0.92														
3. Controlled motivation	2.29	0.89	−0.20***													
4. Amotivation	1.81	0.9	−0.23***	−0.43***												
5. Intrinsic aspirations	3.52	0.85	−0.03	0.49***	0.08											
6. Extrinsic aspirations	2.96	0.85	−0.04	0.24***	0.25***	0.04										
7. Autonomy satisfaction	3.57	0.78	0.13**	0.51***	−0.16***	−0.25***	0.41***									
8. Autonomy frustration	2.45	1	−0.10*	−0.15**	0.57***	0.48***	−0.05	0.12**								
9. Relatedness satisfaction	3.55	0.91	0.03	.20***	-0.04	-.12**	.27***	.13**	.50***							
10. Relatedness frustration	2.06	0.92	−0.11**	−0.05	0.42***	0.40***	0.01	0.10*	−0.12**	0.59***						
11. Competence satisfaction	3.6	0.8	0.09	0.42***	−0.15***	−0.24***	0.30***	0.19***	0.59***	−0.20***	0.45***					
12. Competence frustration	2.61	1.05	−0.10*	−0.03	0.40***	0.28***	0.05	0.02	−0.08	0.59***	−0.12**	0.60***				
13. General wellbeing	3.49	0.82	0.05	0.15***	−0.09*	−0.08	0.20***	0.22***	0.35***	−0.27***	0.46***	−0.34***	0.44***			
14. Supportive relationships	3.92	1.05	0.17***	0.10*	−0.12*	−0.07	0.18***	0.08	0.26***	−0.16***	0.30***	−0.22***	0.19***	−0.13**		
15. Contextual barriers	2.34	0.89	−0.08	−0.05	0.33***	0.29***	−0.02	−0.02	−0.10*	0.55***	−0.16***	0.54***	−0.19***	0.53***	−0.43***	

*p < 0.05, **p < 0.01, ***p < 0.001. *^a^*Completion rates (%) were measured in Time 2 while all other variables were measured in Time 1.

A first exploration highlights the very skewed distribution of the completion rates (%) variable, calculated as the ratio of the number of modules completed relative to the total number of modules enrolled. In this study, 151 students attained a 100% completion rate, hence indicating a ceiling effect, diminishing variance in this dependent variable. Nevertheless, four out of 14 Pearson correlations were significant at *p* < 0.05, with moderate effect sizes (*r* varied between −0.23 and 0.17). There was a significant positive correlation between completion rates and autonomous motivation (*r* = 0.12, *p* = < 0.01), autonomy satisfaction (*r* = 0.13, *p* = < 0.01), and supportive relationships (*r* = 0.17, *p* = < 0.001). Furthermore, there was a negative correlation between completion rates and controlled motivation (*r* = −0.20, *p* = < 0.001), amotivation (*r* = −0.23, *p* = < 0.001), autonomy frustration (*r* = −0.10, *p* = < 0.05), relatedness frustration (*r* = −0.11, *p* = < 0.05), and competence frustration (*r* = −0.10, *p* = < 0.05). The results indicated that the correlation between completion rates and intrinsic aspirations *r* = −0.03), extrinsic aspirations (*r* = −0.04), relatedness satisfaction (*r* = 0.03), competence satisfaction (*r* = 0.09), contextual obstacles (*r* = −0.08), and general wellbeing (*r* = 0.05), were not significant.

Second, further exploration of the descriptives and cross-correlations of our selected variables in Table 6 adds some interesting observations. First, scrutinizing the means and standard deviations indicate that—as a group—drop-in students display substantially higher mean levels in “adaptive” variables (i.e., higher quality motivation indexed by autonomous and intrinsic motives and aspirations, need satisfaction, and general wellbeing) than in the more “maladaptive” variables (i.e., controlled or amotivation, extrinsic aspirations, need frustration, and contextual barriers). Notably, amotivation and (general) relatedness frustration show the lowest mean levels, whereas autonomous motivation, intrinsic aspirations, need satisfaction, and general wellbeing all average around 3.6, measured on a Likert scale from 1 to 5. However, for all variables, large standard deviations are found (ranging from.78 to 1.05), indicating that considerable heterogeneity exists within this group of drop-in students.

Third, inquiring about the correlations concurrently measured at Time 1 also yields some notable findings about the nomological network among individual and environmental variables, such as contextual barriers. Regarding this interplay, we notice that autonomous motivation is only modestly associated with controlled motivation (*r* = −0.13) or general wellbeing (*r* = 0.15), whereas larger correlations are found with intrinsic aspirations (*r* = 0.49), autonomy (*r* = 0.51) and competence (*r* = 0.42) satisfaction. Notably, controlled motivation is only marginally related to general wellbeing (*r* = −0.09), yet it is substantially related to more need frustration in all three needs (*r*s from.40 to.57) and the report of more contextual barriers (*r* = 0.33). The general wellbeing of drop-in students at Time 1 was most closely linked to higher levels of satisfaction in all three needs (*r*s range from 0.35 to 0.46), and less reported contextual barriers (−0.43) and experiences of need frustration (*r*s range from −0.27 to −0.38).

#### 3.2.3 Group differences in continuous independent variables

**FIGURE 3:**
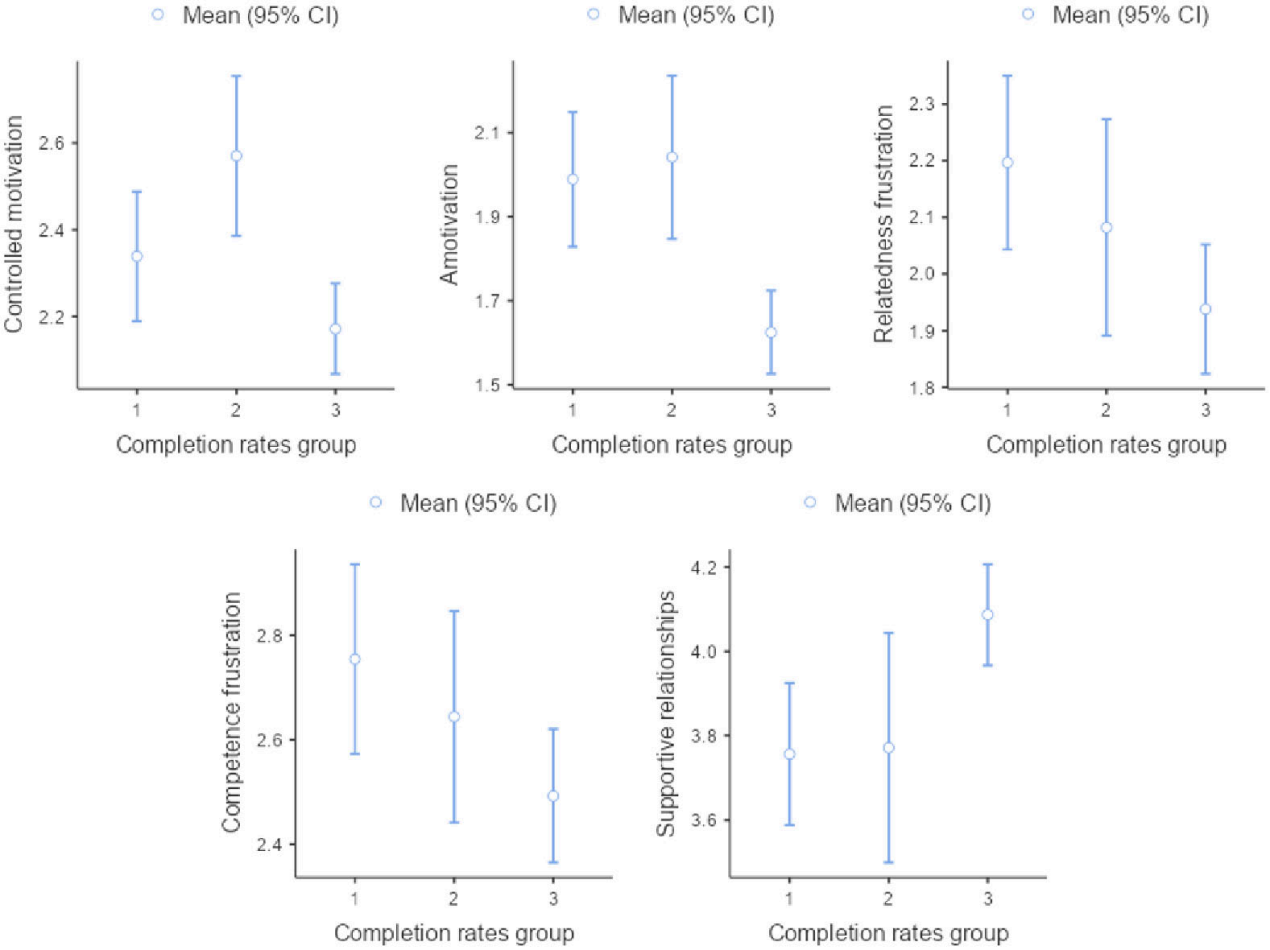
Overview of the significant descriptive plots. Group 1—“low completion rates group” (7V = 162; completion rate > 50.00%), Group 2—“medium completion rates group” (7V = 89; completion rate > 50.00% and < 83.33%), and Group 3—“high completion rates group” *(N* = 253; completion rate > 83.33%).

A one-way MANOVA was conducted to evaluate group differences between the three completion rates groups in the continuously measured set of intrapersonal and environmental variables. Table 7 provides an overview of the MANOVA test, indicating significant group differences, *F*(28, 896) = 2.045, *p* = 0.001; *Wilk’s Lambda* = 0.883. Five major group differences were found in subsequent variance analyses. Groups differed significantly in controlled motivation, *F*(2, 461) = 6.85, *p* = < 0.001, with students in Group 2 scoring significantly higher controlled motivation than students in Group 3, but surprisingly no difference was found between Groups 1 and 3. Notably, students of both Groups 1 and 2 scored significantly higher in amotivation than students in Group 3 [*F*(2, 461) = 11.91, *p* = < 0.001]. Two other group differences involved basic psychological need frustration in relatedness [*F*(2, 461) = 2.96, *p* = < 0.05] and competence [*F*(2, 461) = 3.06, *p* = < 0.05]. Students in Group 3 showed significantly lower frustration in these two basic psychological needs than students in Group 1, yet both groups did not differ significantly from students in Group 2. A final group effect was found for reported supportive relationships [*F*(2, 461) = 5.85, *p* = < 0.001]. Notably, students in both Group 1 and 2 reported less supportive relationships than students in Group 3, yet only the difference between Group 1 and 3 was significant. Figure 3 provides a visualization of the between-group differences of the significant tests.

**TABLE 7:** Overview of MANOVA results between the completion rate groups and the continuous individual and environmental variables.

Variables	Low completion rates	Medium completion rates	High completion rates	*F*-value	*p*-value	Eta-squared
Autonomous motivation	3.50 A	3.49 A	3.69 A	2.76	0.06	0.01
Controlled motivation	2.34 AB	2.57 B	2.17 A	6.85	<0.001 ***	0.03
Amotivation	2.00 B	2.04 B	1.62 A	11.91	<0.001***	0.05
Intrinsic aspirations	3.53 A	3.49 A	3.51 A	0.07	0.94	0
Extrinsic aspirations	2.91 A	3.03 A	2.95 A	0.53	0.59	0
Autonomy satisfaction	3.57 A	3.39 A	3.62 A	2.73	0.07	0.01
Autonomy frustration	2.47 A	2.56 A	2.39 A	1.05	0.35	0
Relatedness satisfactions	3.51 A	3.48 A	3.58 A	0.5	0.61	0
Relatedness frustration	2.19 B	2.08 AB	1.96 A	2.96	<0.05*	0.01
Competence satisfaction	3.53 A	3.57 A	3.65 A	1.18	0.31	0
Competence frustration	2.75 B	2.64 AB	2.49 A	3.06	<0.05*	0.01
General wellbeing	3.39 A	3.45 A	3.56 A	2.04	0.13	0.01
Supportive relationships	3.76 A	3.77 A	4.09 B	5.85	<0.001***	0.02
Contextual barriers	2.46 A	2.41 A	2.25 A	2.79	0.06	0.01
Wilks’ Lambda = 0.883, *F*(28, 896) = 2.045, *p* = 0.001						

The categorical predictor has more than two levels, a multicomparison tukey *post hoc* analysis is added to the table in letters. *p < 0.05, ***p < 0.001.

## 4 Discussion

To the best of our knowledge, this study is one of the first to explore (1) the profiles of drop-in students in SCE, and (2) the processes that could potentially be related to completing educational courses in SCE. To guide this exploration, we built a novel model based upon the large literature on regular (first chance) education and school dropout on the one hand, and the scant studies on Second Chance Education on the other, attempting to address a comprehensive set of variables at the background, individual, and environmental level.

### 4.1 RQ1: who are these students? The profiles of drop-in students enrolling in SCE

To answer this research question, we focused on participants’ background characteristics. Most students in our SCE sample are young, with 71% under 25, slightly younger than prior data (Flemish Department of Education, 2022; Glorieux et al., 2011). About 15% more men than women participated, diverging from official data showing more women in SCE (Flemish Department of Education, 2022) but aligning with higher male dropout rates (Eurostat, 2023).

Around one-third of students speak another language at home or were born outside Belgium, indicating a strong migration background, consistent with SCE literature (Dahlstedt and Fejes, 2019) and higher dropout among non-Dutch speakers (Statistics, 2023). Half of drop-in students experienced grade retention in regular education, versus 4% annually in Flanders (Talloen et al., 2020), highlighting its link to disengagement (Anderson et al., 2019) and the need to reduce retention to improve learning outcomes. Most students have low socio-economic status (SES), limited education, and unstable income (Zorbaz and Ozer, 2020), reflecting typical SCE demographics (Glorieux et al., 2011) and SCE’s mission to support disadvantaged groups (Bühler-Niederberger et al., 2023).

Unexpectedly, the largest group dropped out of general (academic) tracks rather than the expected vocational tracks (BSO, DBSO, dual learning) (Statbel, 2023), likely due to unrecognized foreign qualifications or academic lag prompting a shift to SCE. Students from Special Needs Education were also highly represented; many seek SCE not by choice but to obtain a recognized ISCED level 3 diploma unavailable in their previous track.

### 4.2 RQ2: which processes are potentially related to completion rates of courses of drop-in students in SCE?

Our study is unique in exploring profile characteristics and linking them to “study success” in SCE over time. Assessing academic progress proved more complex than in first chance education, as SCE lacks conventional numerical measures. Students follow tailored modular programs, evaluated continuously rather than through formal exams, and receive only pass/fail qualifications per module. We therefore analyzed completion rates in two ways. First, we examined their distribution and found many participants demonstrated “study success”: about 30% (*N* = 151) completed 100% of modules, and over 50% passed more than 83.33% of enrolled courses. To explore variation in completion, we constructed three groups based on 25-50-75 percentiles (low, medium, high) and conducted group comparisons and correlations with the dimensional completion rate (%).

#### 4.2.1 Group differences in categorical background variables

Interestingly, only a limited number of significant group differences and correlations emerged. First, Dutch-speaking students were more likely to belong to Group 3 (high completion rates) compared to students speaking another language at home. This aligns with literature showing that non-Dutch speakers are over-represented in dropout statistics (Statbel, 2023). Language is crucial for completing course modules, as students must adequately understand instructions and assignments (Sprong and Skopek, 2022). Consequently, language support for students speaking another language at home could be beneficial. Effective measures may include extra language lessons, adaptation of instructional strategies to a multilingual context, translation of study content, or incorporating students’ home language in education (Ziegenfuss et al., 2014; Resch et al., 2023), while fostering a positive teacher attitude (Nakamura et al., 2023).

Second, students without prior grade retention were more likely to belong to Group 3 than those who experienced retention. This is significant because grade retention is a strong predictor of underachievement, truancy, disengagement, and dropout (Keppens and Spruyt, 2018; Anderson et al., 2019). Experiences during first chance education strongly influence second-chance outcomes (Ross and Gray, 2005; Van Den Berghe et al., 2024a). While SCE cannot directly change students’ previous experiences, grade retention appears to be an important “red flag,” warranting further research to understand underlying mechanisms and guide support strategies (Kearney et al., 2022). Policymakers should aim to reduce grade retention by limiting punitive and exclusionary measures that force school mobility (Marchbanks et al., 2013), reconsidering early tracking, implementing common curricula for all students, creating homogeneous classes based on ability, or offering flexible courses for heterogeneous groups to prevent retention among low-achieving students (Keppens and Spruyt, 2018).

#### 4.2.2 Correlations and group differences in individual and environmental variables

Both Pearson correlations and MANOVA tests were conducted to explore associations between individual and environmental variables and completion rates across the three groups (low, medium, high). Five significant group differences emerged.

First, Group 2 scored significantly higher than Group 3 on “controlled motivation,” a type of motivation negatively correlated with completion rates, while “autonomous motivation” showed a positive correlation. This aligns with prior research showing that controlled motivation predicts lower academic performance, persistence difficulties, and reduced wellbeing (Jeno et al., 2018; Ryan, 2023). Interestingly, no significant differences were observed between Groups 1 and 3, consistent with findings that adult learners often exhibit higher controlled or extrinsic motivation due to their goal-oriented focus, such as obtaining a diploma (Kalenda and Kocvarova, 2021; Van Den Berghe et al., 2024a; Gardner et al., 2022).

Second, Groups 1 and 2 showed significantly higher “amotivation” compared to Group 3, with the strongest negative correlation observed between amotivation and completion rates. This supports literature identifying amotivation as a major risk factor for underachievement and dropout (Ryan, 2023; Pikkarainen et al., 2021). These first two findings collectively suggest that lower-quality motivation—either controlled or absent—may underlie reduced academic achievement (Ryan, 2023; Archambault et al., 2022).

Third and fourth, regarding basic psychological needs, Group 1 reported significantly higher “relatedness frustration” and “competence frustration” than Group 3, both negatively correlated with completion rates. In contrast, need satisfaction measures (“relatedness,” “competence,” and “autonomy” satisfaction) showed no significant group differences, although autonomy satisfaction had a small positive correlation. This pattern indicates that need frustration may exert a stronger influence on completion rates than need satisfaction. It is possible that most students reported their psychological needs at the beginning of enrollment, limiting the likelihood of capturing satisfaction experiences, particularly in competence. Previous research emphasizes the importance of supporting students who experienced school failure to “finish unfinished business” through SCE (Morelli et al., 2023; Hickey et al., 2020; Gueta and Berkovich, 2021; Savelsberg et al., 2017; Van Den Berghe et al., 2024a; Pikkarainen et al., 2021).

Fifth, Groups 1 and 2 reported significantly lower “supportive relationships” outside the educational context compared to Group 3, with a positive correlation between supportive relationships and completion rates. This finding aligns with evidence that limited family or peer support increases the risk of dropout, truancy, and non-completion (Lawrence and Adebowale, 2023; Virtanen et al., 2022). For drop-in students, building social networks is crucial for engagement in educational and employment pathways, highlighting the relevance of mentoring and social support within SCE programs (Savelsberg et al., 2017; Ross and Gray, 2005).

Together, these results underscore the importance of addressing both motivational quality, psychological need frustrations, and social support systems to enhance completion rates in SCE students, providing clear targets for educators and policymakers.

### 4.3 Insights from this study for supporting students in education

This study offers at least four key insights for future research and practice in supporting students in SCE. First, the study highlights the heterogeneous profiles of drop-in students in Flanders, Belgium (cf. RQ1). Descriptive analyses and cross-correlations revealed considerable variation across all variables, indicating the need for further research to understand differences in developmental and educational outcomes within this group. This aligns with prior studies showing that students with school attendance problems or those who drop out represent a highly diverse population influenced by multiple ecological factors (Gubbels et al., 2019; Duygu et al., 2024). Drop-in students in SCE are part of this population, having previously experienced dropout themselves. Initially, SCE targeted adults with low literacy and numeracy skills, but changing student demographics have shifted its mission, expectations, and focus (Van Eck and Buisman, 2018).

Second, the population of students in SCE has changed over time, with each drop-in student bringing a unique combination of ecological and personal factors (cf. RQ1). These include language differences, younger ages than typically encountered by teachers, and special educational needs. Post-COVID-19, a global learning crisis is anticipated, with students facing challenges in catching up, fatigue, absenteeism, and higher academic failure (UNESCO et al., 2023; Havik and Ingul, 2022). As dropout rates continue to rise, the drop-in student population will likely evolve further, requiring SCE to adapt educational designs, teaching strategies, and lesson content while promoting equity and social inclusion (Bühler-Niederberger et al., 2023; Vanderavero et al., 2025). Understanding the complex interplay of background, individual, and environmental factors related to dropout and drop-in processes can inform meaningful student support (Gren-Landell, 2021; Heyne et al., 2020; Melvin et al., 2019; Moreno and Támara, 2024).

Third, constructs affecting completion rates were explored (cf. RQ2). Educational motivation emerged as central, suggesting various ways to support SCE students: promoting understanding and classroom management (Anderman et al., 2011), investing in school attendance teams (Heyne, 2024), building teacher–student relationships (Leenknecht et al., 2023), addressing beliefs about amotivation (Banerjee and Halder, 2021), incorporating relevant activities (Radil et al., 2023), and fostering autonomy-supportive climates (Gueta and Berkovich, 2021). Basic psychological needs, supportive relationships, and emotional engagement were also crucial (Hope et al., 2019; Van Den Berghe et al., 2022a). Motivation alone, however, does not fully predict completion; students’ wellbeing and life circumstances—housing, finances, family obligations, health—can prevent motivated students from translating intention into academic behavior (Masten, 2014; Kaya and Erdem, 2021; Morelli et al., 2023; Portela Pruaño et al., 2022; Gren-Landell, 2021).

Finally, life course events and social context can make “school secondary” (Van Den Berghe et al., 2022b), frustrating psychological needs and lowering wellbeing, ultimately reducing completion rates (Ryan, 2023; Vanderavero et al., 2025). Supporting students in SCE thus requires recognizing broad benefits of education beyond economic outcomes, including personal development and social skills (te Riele, 2011; Sureda-Garcia et al., 2025). Holistic approaches that consider the interconnectedness of life domains and intersectional aspects of students’ narratives are essential. Prioritizing both educational motivation and wellbeing aligns with Biesta’s principle that education must ensure quality learning opportunities for all (Biesta, 2015, p. 81).

### 4.4 Limitations of this study and implications for future research

The generalizability of this study’s results is constrained by several limitations. First, aside from language spoken at home and prior grade retention, no significant relationships were found between background characteristics and belonging to a completion rates group, whereas prior research identified factors such as age, gender, and type of education before dropout as influential (Piscitello et al., 2022; Eurostat, 2023). It is possible that the phrasing of questions and coding of variables introduced bias. Future research aiming to explore background characteristics affecting course completion among drop-in students would benefit from using control groups and a more focused analysis on these variables.

Second, the Dutch versions of the questionnaires were used, although 39.7% (*N* = 210) of participating students (*N* = 528) spoke another language at home. Language comprehension issues may have affected responses. Additionally, self-designed items were used to measure constructs such as supportive relationships in education. Despite pilot testing and high scale reliability, these measures remain a limitation of the study.

Third, although students were selected at random, recruitment procedures may have introduced sample bias. Replicating these results ideally requires comparison with control groups, which is practically challenging due to difficulties in reaching participants and the inherent heterogeneity of SCE populations (Van Den Berghe et al., 2024a).

Fourth, using completion-rate groups for comparisons introduces potential pitfalls. Categorical grouping is less sensitive than continuous measures, as group membership is binary (“yes” or “no”) (Agresti, 2013). This may partly explain why no significant differences were observed for constructs such as “educational aspirations” or “contextual barriers,” despite theoretical expectations.

Nevertheless, despite these limitations, this study provides a valuable exploration in a field with limited research. It examines the diverse and heterogeneous population of drop-in students in Flanders, Belgium, highlighting the potential influence of background characteristics, educational motivation, aspirations, basic psychological needs, support and barriers in education, and general wellbeing on course completion in SCE. By doing so, it opens pathways for future research and the development of targeted support strategies to enhance learning outcomes for all students.

## Data Availability

The original contributions presented in this study are included in this article/supplementary material, further inquiries can be directed to the corresponding authors.
